# 间充质干细胞对非小细胞肺癌细胞增殖和侵袭能力的初步探讨

**DOI:** 10.3779/j.issn.1009-3419.2015.11.03

**Published:** 2015-11-20

**Authors:** 梅 李, 毅 武, 仁旺 刘, 丽丽 郭, 婷婷 徐, 军 陈, 嵩 徐

**Affiliations:** 1 300052 天津，天津医科大学总医院肺部肿瘤外科 Department of Lung Cancer Surgery, Tianjin Medical University General Hospital, Tianjin 300052, China; 2 300052 天津，天津医科大学总医院，天津市肺癌研究所 Tianjin Key Laboratory of Lung Cancer Metastasis and Tumor Microenvironment, Tianjin Lung Cancer Institute, Tianjin Medical University General Hospital, Tianjin 300052, China; 3 300070 天津，天津医科大学肿瘤医院 Tianjin Medical University Cancer Hospital, Tianjin 300060, China

**Keywords:** 肺肿瘤, 间充质干细胞, 趋化, 增殖, Lung neoplasms, Mesenchymal stem cells, Migration, Proliferation

## Abstract

**背景与目的:**

间充质干细胞(mesenchymal stem cells, MSC)是来源于中胚层的成体干细胞。有文献报道MSC通过向肿瘤组织的归巢和向间质成分分化，改变肿瘤微环境，影响肿瘤的生长和转移。但MSC在非小细胞肺癌(non-small cell lung cancer, NSCLC)中的作用报道较少，且不一致。本研究旨在探讨MSC向NSCLC细胞的趋化能力，以及其对NSCLC细胞的增殖和侵袭能力的作用。

**方法:**

Transwell法检测MSC向肺癌细胞迁移能力，Thymidine嵌入实验检测MSC条件培养液对肺癌细胞增殖能力的影响，Real-time PCR法检测肺癌/MSC共培养后MSC表达白介素(interleukin-6, IL-6)、胰岛素样生长因子(insulinlike growth factor, IGF-1)、血管内皮生长因子(vascular endothelial growth factor, VEGF)和Dickkopf相关蛋白1(dickkopf-related protein 1, DKK1)的变化。建立人肺癌A549细胞裸鼠皮下荷瘤模型，给予MSC细胞，定期测量肿瘤体积变化。

**结果:**

MSC可以向肺癌细胞趋化运动，其条件培养液可以促进肺癌细胞的增殖能力。肺癌细胞反过来可以促进MSC过表达IL-6、IGF-1、VEGF和DKK1。体内试验显示MSC注射组的肿瘤体积明显大于对照组。

**结论:**

MSC可以向肺癌细胞趋化并促进肺癌的生长。反过来，肺癌细胞可以刺激MSC过表达生长因子进一步促进肿瘤生长。

## Abstract (en)

**Background and objective:**

Mesenchymal stem cells (MSC) are adult stem cells derived from mesoderm. Evidence has shown that MSC could migrate towards tumor tissue and differentiate into tumor associated fibroblast in tumor microenvironment, which influences tumor growth and metastasis. However, the reports of MSC in non-small cell lung cancer (NSCLC) are few and controversial. The aim of this study is to explore the chemotaxis of MSC towards NSCLC and to test the effects of MSC on the proliferation and invasion ability of NSCLC.

**Methods:**

Transwell assay was used to test MSC and NSCLC migration and invasion, and Thymidine incorporation assay was adopted to measure NSCLC cells proliferation. The expression of interleukin-6 (IL-6), insulinlike growth factor (IGF-1), vascular endothelial growth factor (VEGF) and dickkopf-related protein 1 (DKK1) of MSCs were determined by real time PCR. A549 lung cancer xenograft animal tumor model was set up to evaluate the MSC effect *in vivo*.

**Results:**

Lung cancer cells could attract MSC tropism. MSC conditioned medium favored lung cancer cell proliferation and lung cancer cells stimulated the expression of IL-6, IGF-1, VEGF and DKK1 on MSCs. *In vivo* animal study showed that the tumor with MSC injection grew much faster compared to control group.

**Conclusion:**

MSCs could migrate towards NSCLC cells and favor tumor growth. In turn, NSCLC cells could stimulate the overexpression of cytokines on MSCs which are essential for the tumor growth.

骨髓间充质干细胞(mesenchymal stem cells, MSC)是来源于中胚层的成体干细胞，具有自我更新、增殖能力。MSC是存在于骨髓中的多潜能干细胞。他在适宜的信号刺激下能够诱导分化成不同的骨髓微环境的组成成分，如骨、脂肪、造血和基质细胞。MSC能够分泌许多造血因子，包括巨噬细胞集落刺激因子(macrophage colony-stimulating factor, M-CSF)、白介素(interleukin-6, IL-6)、SCF和白血病抑制因子(leukemia inhibitory factor, LIF)等来支持造血。此外，由于MSC的免疫抑制作用和多向分化潜能，MSC还被应用于器官移植和组织修复等方面。

目前MSC与肿瘤细胞之间关系的研究越来越受到关注。有文献^[1, 2]^报道MSC其具有靶向迁移至肿瘤部位的特性。通过向肿瘤组织的归巢和向间质成分分化，改变肿瘤微环境，影响肿瘤的生长和转移。我们的研究旨在探讨肿瘤微环境中的未经修饰的MSC在非小细胞肺癌(non-small cell lung cancer, NSCLC)的侵袭和转移方面的功能研究。但是，NSCLC来源MSC的生物学性状和MSC在NSCLC发生发展中的作用的相关研究报道的结果很不一致，阐述的机制也不清楚。

## 1 材料与方法

### 1.1 MSC细胞培养

骨髓取自正常捐献者，签署知情同意书。应用Ficoll-Hyaque密度梯度离心后提取单核细胞，PBS洗涤后混于MesenPro培养液(Gibco)，含有2%胎牛血清，1%抗生素，2%MesenPro生长添加物，调整细胞密度1×10^6^/cm^2^的置于细胞培养瓶后，细胞培养箱过夜。PBS洗去未贴壁细胞，更换新的MesenPro培养液。每2-3天换液，5 d-7 d后细胞融合至80%左右(即第1代)，应用胰酶消化细胞，按照细胞密度2×10^3^/cm^2^置于新的细胞培养瓶。重复传代。第2代和第3代MSC应用于本研究。用于本实验前，应用流式细胞鉴定MSC纯度，CD90(+)，CD73(+)，CD105(+)，CD45(-)，同时具有成纤维细胞形态，并向脂肪，成骨和软骨分化能力。

### 1.2 3H胸腺嘧啶核苷掺入法

1×10^4^ NSCLC细胞置于96孔板过夜，16 h后吸除培养液，PBS洗一遍后加入MSC条件培养液，再培养24 h。NSCLC细胞的增殖能力通过3H胸腺嘧啶核苷掺入法测定相对DNA合成指数与未经过MSC条件培养液暴露的NSCLC细胞做对比。

### 1.3 Transwell体外迁移实验

应用Costar公司的Transwell小室做体外迁移实验。无血清培养液，10%小牛血清，或NSCLC细胞置于下室作为吸引物，上室放置MSC细胞，24 h后计数Tranwell上室膜的MSC数量。此外，CCR9敲除的MSC重复此迁移实验。

### 1.4 RNA干扰CCR9表达试验

为敲除CCR9表达，MSC转染FlexiTube GeneSolution for CCR9 (GS10803, Qiagen, Hilden, Germany, www.qiagen.com)。FlexiTube GeneSolution for CCR9包含4对互不重叠的CCR9RNAi链。Lipofectamine RNAiMAX(Invitrogen)作为转染剂。AllStarsNegative Control small interfering RNA(SI03650318, Qiagen)作为阴性对照。实时定量PCR和流式细胞检测CCR9敲除效率达到90%以上。

### 1.5 MSC和NSCLC细胞共培养试验

MSC铺Transwell培养板(0.4 μM孔径，Corning，NY，USA)下室过夜，待MSC贴壁后于Transwell上室放置NSCLC细胞(MSC:NSCLC细胞=1:10)，共培养72 h后胰酶消化收集MSC细胞，提取RNA备用。

### 1.6 MSC条件培养液的制备

2.5×10^5^ MSC培养于5 mL无血清RPMI-1640培养液48 h，转移培养液至离心管离心2, 000 rpm，吸取上清液，冻存-20 ℃备用。

### 1.7 实时定量PCR

应用Trizol法提取RNA，Thermoscript reverse-transcription PCR system用于逆转cDNA，实时定量PCR分析使用iCycler(Bio-Rad Laboratories, Hercules, CA)和BR GreenER qPCR SuperMix for iCycler(Invitrogen)。引物序列如下所示：IL-6，F：5’-TCTCCACAAGCGCCTTCG-3’；R：5’-CTCAGGGCTGAGATGCCG-3’；DKK1, F：5’-GATCATAGCACCTTGGATGGG-3’；R：5’-GGCACAGTCTGATGACCGG-3’；胰岛素样生长因子(insulin like growth factor, IGF-1)和血管内皮生长因子(vascular endothelial growth factor, VEGF)引物购自Realtimeprimers.com；内参为beta-actin：F：5'-ATGTGGCCGAGGACTTTGATT-3'；R：5'-AGTGGGGTGGCTTTTAGGATG-3'。2^-△△ct^用于分析相对表达水平。

### 1.8 皮下荷瘤模型

6周龄裸鼠购自中国医学科学院肿瘤研究所。取对数生长期NSCLC细胞A549和MSC。A549细胞(1×10^8^)注射于裸鼠右腿与腹腔临近部位，待肿瘤生长约200 mm^3^时，分成两组，每组各10只。第一组瘤体内注射生理盐水，第二组瘤体注射MSC细胞(1×10^6^ MSC)，每隔3天打一次，共注射3次。裸鼠置于恒温(25±2)℃，恒湿(45%-50%)的无菌净化屏障系统内饲养，每隔3天测量瘤径，观察记录肿瘤生成和生长情况，游标卡尺测量肿瘤结节的最长径a和最短径b，肿瘤体积计算公式如下：V=(ab^2^)/2，求其平均值。绘制肿瘤生长曲线。

### 1.9 统计学方法

所有实验至少重复3次。采用软件GraphPad Prism 5分析数据，选用方差分析和*t*检验，*P*＜0.05为差异有统计学意义。

## 2 结果

### 2.1 体外migration assay提示NSCLC细胞可以吸引MSC迁移

MSC可以向肿瘤细胞定向运动和迁移，已经在很多肿瘤模型中得到了证实。我们之前工作发现骨髓瘤细胞可以吸引MSC迁移，并确定CCL25/CCR9轴是导致MSC迁移的重要机制^[3]^。这里，我们应用体外Transwell培养体系，证实NSCLC细胞同样可以吸引MSC的迁移，提示MSC可以从骨髓动员向肿瘤部位运动，组成或分化成为NSCLC相关间质，影响NSCLC细胞各种恶性表型。此外，我们发现MSC的CCR9表达敲低后，MSC向NSCLC细胞A549和L9981的迁移能力下降了35.8%和20.1%，具有统计学差异。提示CCR9在MSC向NSCLC迁移中具有重要作用(图 1)。

**1:**
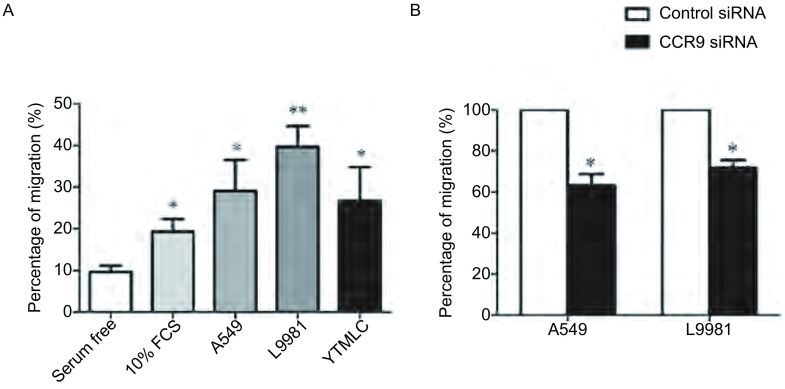
NSCLC细胞可以吸引MSC迁移。A：Transwell培养系统显示MSC具有向NSCLC细胞迁移的能力。无血清和10%胎牛血清培养液作为对照；B：CCR9表达敲低的MSC向NSCLC细胞迁移能力降低。^*^P＜0.05；^**^P＜0.01。 Non-small cell lung cancer (NSCLC) cells could attract mesenchymal stem cells (MSC) migration. A: MSC migrated towards NSCLC cells in transwell culture system. Serum free medium and 10% FCS medium as negative and positive control; B: CCR9 knockdown MSC showed decreased migration ability towards NSCLC cells. ^*^: Compared with the serum free group or control siRNA group, *P* < 0.05; ^**^: Compared with the serum free group, *P* < 0.01.

### 2.2 NSCLC细胞可以刺激MSC上调表达生长因子和细胞因子

肿瘤细胞和肿瘤间质细胞之间是个动态的交互过程。在与肿瘤细胞的交互过程中，正常的间质细胞“学习”到了某些异常的特征，从而提供了适合肿瘤生长的微环境，例如，相比正常间质细胞，肿瘤相关间质细胞可以高表达某些与肿瘤生长相关的生长因子和/或细胞因子。我们应用体外Transwell培养体系共培养MSC和NSCLC细胞A549和L9981 48 h后，发现MSC表达IL6、IGF-1、VEGF和DKK1明显升高，提示肺癌细胞和MSC之间通过旁分泌存在一个反馈调节机制(图 2)。

**2:**
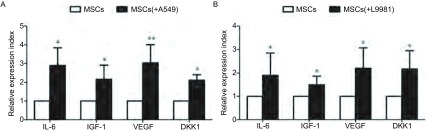
Real Time PCR检测发现NSCLC细胞刺激MSC高表达IL-6、IGF-1、VEGF和DKK1。A：A549细胞；B：L9981细胞。^*^P＜0.05；^**^P＜0.01. Real time PCR test showed that NSCLC cells stimulated interleukin-6 (IL-6), insulinlike growth factor-1 (IGF-1), vascular endothelial growth factor (VEGF) and dickkopf-related protein 1 (DKK1) expression on MSCs. A: A549 cells; B: L9981 cells. ^*^: Compared with MSC alone group, *P* < 0.05; ^**^: Compared with MSC alone group, *P* < 0.01.

### 2.3 MSC条件培养液可以促进NSCLC细胞的增殖和侵袭迁移能力

我们用MSC条件培养液来培养NSCLC细胞1 d和3 d。3H胸腺嘧啶核苷掺入法检测发现MSC条件培养液培养NSCLC细胞3 d后，肿瘤细胞的增殖能力明显升高(培养1 d后肿瘤细胞增殖能力升高不明显，结果未示)。此外，应用Transwell培养体系，将肺癌细胞置于上室，上室铺上Matrigel胶模拟细胞外基质，同时上室添加MSC条件培养液，下室用10%小牛血清作为趋化物。24 h后，我们发现暴露于MSC条件培养液的肿瘤细胞的迁移能力明显升高(因为MSC条件培养液培养NSCLC细胞24 h后，NSCLC细胞的增殖能力变化不明显，所以可以排除NSCLC细胞数目增加导致的迁移能力增强)。这些结果提示MSC确实通过旁分泌机制可以促进NSCLC细胞生长，并且可能参与促进NSCLC细胞的侵袭和转移(图 3)。

**3:**
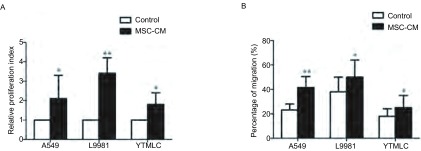
MSC条件培养液促进肺癌细胞增殖和侵袭迁移能力。A：3H胸腺嘧啶核苷掺入法用于NSCLC细胞增殖能力；B：Transwell培养体系用于NSCLC细胞迁移能力检测。^*^P＜0.05；^**^P＜0.01. MSC conditioned medium stimulated the proliferation and invasion ability of NSCLC. A: 3H thymidine incorporation assay used to test proliferation; B: Transwell coculture system used to test migration. ^*^: Compared with control group, *P* < 0.05; ^**^: Compared with control group, *P* < 0.01.

### 2.4 体内动物实验

在观察期内，MSC注射组的裸鼠肿瘤生长速度明显快于对照组，自第13天开始，肿瘤生长有统计学差异，而且差异持续存在(图 4)。治疗结束时肿瘤体积分别为：对照组：(754.18±294.45)mm^3^，MSC注射组：(1, 223.45±397.34)mm^3^。

**4:**
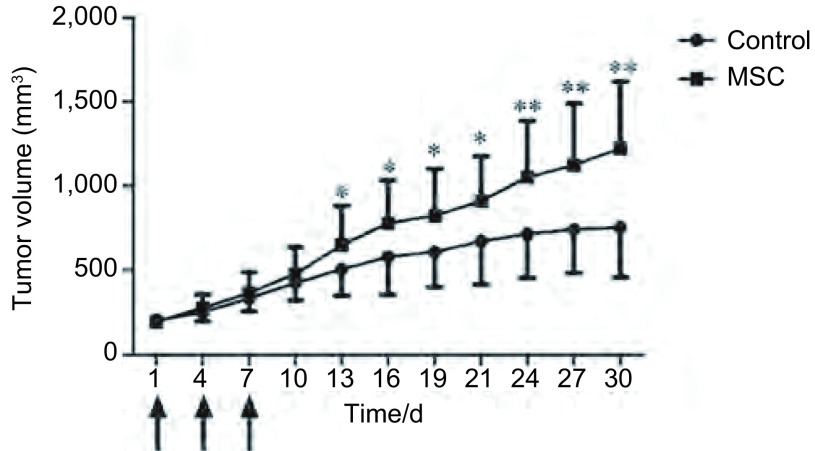
体内动物实验显示MSC注射组的小鼠皮下肿瘤生长明显快于对照组。^*^P＜0.05；^**^P＜0.01。箭头为注射MSC或生理盐水。 In vivo animal study showed that the tumor from MSC injection group grew much faster compared to the saline injection group with statistical significant difference. ^*^: Compared with control group, *P* < 0.05; ^**^: Compared with control group, *P* < 0.01.

## 3 讨论

MSC在多种肿瘤的发生和发展中具有重要作用，是当前肿瘤的研究热点，但是在肺癌方面，相关的报道不是很多。有研究显示NSCLC组织来源的MSC表现不同于正常MSC的生物学性状。2013年Gottschling等^[4]^的研究显示肺癌组织来源的MSC(NSCLC-MSC)的增殖能力高于正常肺组织MSC(normal lung tissue-MSC, NLT-MSC)，NSCLC-MSC有62个基因表达，不同于NLT-MSC，涉及细胞增殖、DNA修复、细胞外基质合成、组织重塑和血管生成等。而且肿瘤细胞的条件培养液可以刺激MSC高表达肿瘤相关成纤维细胞(cancer-associated fibroblasts, CAF)的标志物α-平滑肌肌动蛋白(α-smooth muscle actin, α-SMA)，提示MSC可能是肺癌组织CAF的前体细胞。Fernández等^[5]^研究发现肺癌患者的骨髓MSC表现骨分化和脂肪分化能力降低，CD146的表达也下调，而软骨分化能力升高。这两篇报道提示肺癌患者和肿瘤组织来源的MSC的生物学性状均发生了改变，而这些改变可能影响着肺癌疾病的发生和发展。关于MSC在肺癌生长、转移和耐药等恶性表型的功能学方面的研究，报道并不多，而且相关报道的结果也不太一致。日本学者Suzuki等^[6]^应用Lewis小鼠肺癌模型发现MSC通过增强血管新生而促进肿瘤的生长^[7]^。韩国研究小组也证实在A549小鼠肺癌模型中MSC可以通过溶血磷脂酸受体1(lysophosphatidic acid receptor 1，LPA1)介导的血管生成而促进肿瘤的体内生长。刘峰等^[8]^曾报道骨髓MSCs与Lewis细胞同时接种可加速小鼠肺癌皮下移植瘤形成，而成瘤后MSCs瘤体内注射具有促进移植瘤生长的作用。台湾的一个研究小组也证实了MSC可以增强A549和CL1-5肺癌细胞的体外克隆形成能力和体内皮下成瘤率和瘤体生长能力，并且进一步发现阻断MSC分泌的IL-6可以部分阻断MSC的支持成瘤能力^[9]^。但是，还有一些研究报道MSC可以抑制肺癌细胞的生长，甚至体内和体外表现为相反的作用。卢兆桐等^[10]^发现脐带来源的MSC对C57BL/6小鼠Lewis肺癌本身的生长无影响，但能够抑制肿瘤的肺转移能力。Li等^[11]^也发现在体外MSC抑制肺癌细胞A549和SK-MES-1的增殖，促进其凋亡和侵袭能力。然而，动物体内实验结果与体外实验结果相反，MSCs在体内促进了肿瘤的形成和生长。

我们的研究结果显示，MSC可以向肺癌细胞趋化运动。CCR9敲低表达后，MSC向肺癌细胞迁移能力明显降低，提示CCR9是介导MSC向肺癌细胞移动的重要趋化因子受体。MSC一旦迁移到肺癌肿瘤微环境，就会与肺癌细胞发生交互作用(crosstalk)^[12]^。我们发现MSC可以通过旁分泌机制促进肺癌细胞的增殖，而肺癌细胞反过来又可以刺激MSC高表达IL6、VEGF、IGF-1和DKK1等细胞因子，进一步促进肺癌细胞的生长和侵袭能力。体内动物试验发现MSC注射的裸鼠皮下肿瘤生长速度更快。这进一步验证了MSC对于肺癌细胞的生长具有重要的促进作用。

然而，此研究还有一些需要进一步探讨的地方，比如，MSC到底是通过分泌哪些因子来促进肺癌细胞的生长，具体的调控是什么？或者，肺癌微环境中的MSC异常生物学性状的原因是什么？这些问题需要今后深入研究。

总之，我们研究证实MSC在肺癌肿瘤微环境中不仅仅是“旁观者”(bystander)，而是作为重要的“帮手”(helper)促进肺癌细胞的生长等恶性表型，在肺癌的发生和发展中起到重要的作用。靶向肿瘤微环境MSC，切断其与肿瘤细胞之间的相关作用，可能是今后治疗肺癌的治疗手段。
